# Improving productivity and soil fertility in *Medicago sativa* and *Hordeum marinum* through intercropping under saline conditions

**DOI:** 10.1186/s12870-024-04820-3

**Published:** 2024-03-01

**Authors:** Amal Guerchi, Wiem Mnafgui, Cheima Jabri, Meriem Merghni, Kalthoum Sifaoui, Asma Mahjoub, Ndiko Ludidi, Mounawer Badri

**Affiliations:** 1 Laboratory of Extremophile Plants, Centre of Biotechnology of Borj Cedria, B.P. 901, Hammam-Lif, 2050 Tunisia; 2 Faculty of Sciences of Tunis, University of Tunis ElManar, Campus Universitaire El-Manar, Tunis, 2092 Tunisia; 3 Direction des Sols, INRAT, Rue Hedi Karray, Menzah, 1004 Tunisia; 4 Plant Stress Tolerance Laboratory, University of Mpumalanga, Private Bag X112831, Mbombela, 1200 South Africa; 5 DSI -NRF Centre of Excellence in Food Security, University of the Western Cape, Robert Sobukwe Road, Bellville, 7530 South Africa

**Keywords:** Crop productivity, Soil chemical properties, Salinity, Intercropping, sustainable agriculture

## Abstract

**Background and aims:**

Intercropping is an agriculture system used to enhance the efficiency of resource utilization and maximize crop yield grown under environmental stress such as salinity. Nevertheless, the impact of intercropping forage legumes with annual cereals on soil salinity remains unexplored. This research aimed to propose an intercropping system with alfalfa (*Medicago sativa*)/sea barley (*Hordeum marinum*) to explore its potential effects on plant productivity, nutrient uptake, and soil salinity.

**Methods:**

The experiment involved three harvests of alfalfa and *Hordeum marinum* conducted under three cropping systems (sole, mixed, parallel) and subjected to salinity treatments (0 and 150 mM NaCl). Agronomical traits, nutrient uptake, and soil properties were analyzed.

**Results:**

revealed that the variation in the measured traits in both species was influenced by the cultivation mode, treatment, and the interaction between cultivation mode and treatment. The cultivation had the most significant impact. Moreover, the mixed culture (MC) significantly enhanced the *H. marinum* and *M. sativa* productivity increasing biomass yield and development growth under salinity compared to other systems, especially at the second harvest. Furthermore, both intercropping systems alleviated the nutrient uptake under salt stress, as noted by the highest levels of K^+^/Na^+^ and Ca^2+^/Mg^2+^ ratios compared to monoculture. However, the intercropping mode reduced the pH and the electroconductivity (CEC) of the salt soil and increased the percentage of organic matter and the total carbon mostly with the MC system.

**Conclusions:**

Intercropped alfalfa and sea barely could mitigate the soil salinity, improve their yield productivity, and enhance nutrient uptake. Based on these findings, we suggest implementing the mixed-culture system for both target crops in arid and semi-arid regions, which further promotes sustainable agricultural practices.

**Supplementary Information:**

The online version contains supplementary material available at 10.1186/s12870-024-04820-3.

## Background

The agricultural sector is facing significant challenges due to the increasing global population and its escalating food demands [1]. Successful agricultural systems rely on various elements, including fertilization practices, types of plantations, cropping systems, and a range of biotic and abiotic factors to achieve their goals. Abiotic stresses, as primary constraints, significantly impact soil ecosystems and agricultural output, affecting plant defense mechanisms and physiological responses [2]. These stresses often lead to a 70% reduction in plant products and a 50% decrease in yield [3]. Approximately 20% of agricultural lands worldwide are affected by soil salinization to varying degrees [4], with 3.8% of these lands located in Africa [5]. In Tunisia, where a considerable portion of agricultural areas experiences arid and semi-arid climates, salinity poses severe challenges [6], leading to significant reductions in productivity [7, 8].

To address this challenge, mitigating salinity stress involves two primary approaches: the technological strategy, often associated with higher costs [9], and innovative agricultural practices, such as intercropping systems, recently highlighted in several research studies [10–12]. Intercropping involves cultivating two or more species simultaneously in the same field for a significant part of their growth [13]. Previous research has shown the positive impact of intercropping on soil salinity [14, 15]. It reduces surface soil evaporation and secondary salinization by increasing soil coverage and enhances soil salt solubility through the production of organic acids via root excretion [16]. Intercropping with halophytes or salt-tolerant forage crops has advantages in reducing topsoil salinity, as evidenced in studies showing reduced salt accumulation, improved soil properties, and increased crop productivity in saline soils [17].

Forage crops with deep roots in intercropping systems draw water from deeper soils, reducing salt lift with water and preventing salt accumulation in topsoil [18]. Additionally, salts are removed from the system with forage harvests, offering potential for multiple harvests in a single year [17, 19]. Studies have demonstrated that intercropping systems enhance salinity tolerance, leading to higher forage yield and quality in saline conditions [20].

Legumes/cereals are the most common combination for intercropping [12], playing a crucial role in nitrogen fixation and optimizing nitrogen utilization in plants. These systems promote synergistic mechanisms, sharing soil nutrients, water, and nutrient uptake, reducing dependence on nitrogen fertilizer input and positively impacting intensive agricultural systems.

Alfalfa (*Medicago sativa* L.) is a major forage crop cultivated in Tunisian arid and semi-arid areas [21, 22]. Cultivating alfalfa has the potential to alleviate soil salt accumulation and aid in sodium removal, leading to improved soil pH, enhanced soil porosity, and increased organic carbon content [18]. Intercropping alfalfa with other crops like wheat [23] and maize [24] is becoming increasingly popular. Sea barley (*Hordeum marinum*), a nitrophilic grass well adapted to Tunisian saline conditions, exhibits high salt tolerance [25]. It is instrumental in enhancing biomass productivity and is considered a model for improving salt tolerance in other food crops [25, 26]. However, the impacts of alfalfa intercropped with *Hordeum marinum* on soil alkali-salinity remain inadequately understood.

In our study, we hypothesize that intercropping spring *Medicago sativa* (alfalfa) with *Hordeum marinum* (sea barley) can mitigate soil salt accumulation and related properties while enhancing forage productivity and agronomic traits. Our primary objective is to investigate the impact of intercropping alfalfa with sea barley on plant productivity (agronomic traits) and the status of soil nutrient and physicochemical properties.

## Materials and methods

### Plant material and growth conditions

Throughout this study, we utilized alfalfa (*M. sativa*) Gabes2353, a variety derived from a Tunisian breeding program [21, 22] known for its adaptability to salinity [27]. Currently, it is the predominant cultivar in Southern Tunisia. Alfalfa is typically sown in April, and it can be harvested three times annually. The seeds of the Kl4 line of *H. marinum* were collected from the saline area (Sebkhet El Kalbia) in Kairouan, located in central Tunisia. This Kl4 line was developed through two generations of self-pollination in a greenhouse at the Centre of Biotechnology of Borj Cedria (CBBC). It’s worth noting that *H. marinum* is a predominantly self-pollinating species exhibiting a very high level of homozygosity [25, 28]. The distinct growth dynamics of alfalfa and sea barley facilitate the establishment of temporal and spatial complementarity, promoting efficient utilization of light, water, and nutrients.

Seeds were germinated in Petri dishes on filter paper moistened with distilled water in the dark in an incubator at 25 °C. Seedlings of each variety were transplanted into 50-liter plastic boxes (61.5 × 39.5 × 33 cm) (length/width/height), filled with 33 kg of garden soil mixed with 1800 g of adequately fermented compost obtained from sheep manure residues, applied at a rate of 10 tons per hectare, which is a common agricultural practice in Tunisia among farmers.

The soil studied was classified as a meadow soil which has a pH 8.5, available nitrogen of 13.10 mg kg^− 1^, available phosphorus of 8.19 ppm, and available potassium of 355 ppm. The seedling growth experiment took place at the Centre of Biotechnology of Borj Cedria (CBBC) in Tunisia, situated at a latitude of 36°41′13″ (N) and a longitude of 10°22′55″(E), with an elevation of 70 m above sea level. This location is characterized by a coastal region with a predominant semi-arid climate. The average annual rainfall at this station is 450 mm, and the mean annual temperature was 18.6 °C [29]. The experiment spanned from April to July 2021 and was conducted within a greenhouse setting to eliminate potential interference from rain.

**Fig. 1:**
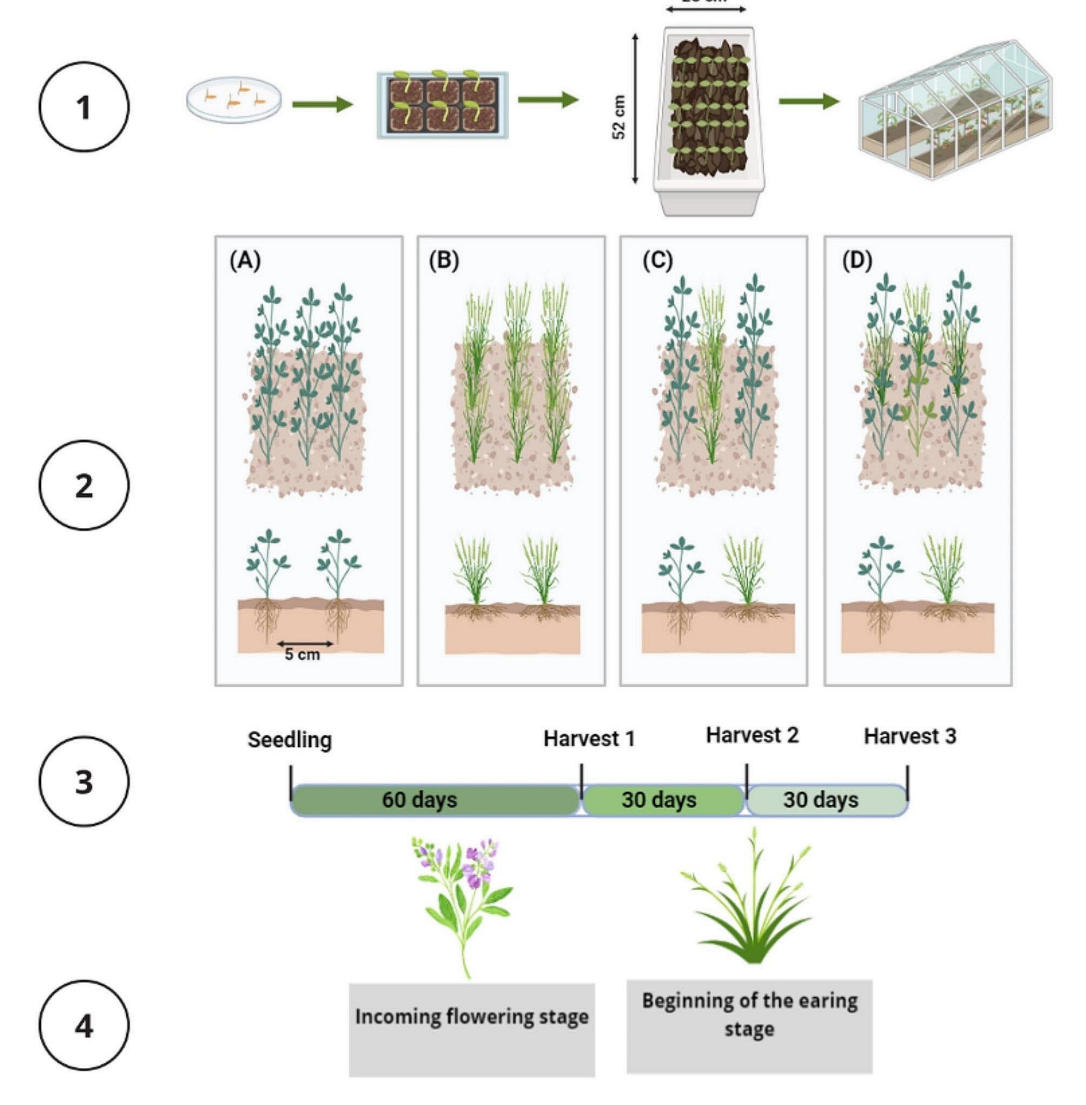
Planting in the greenhouse (1) of *H. marinum* and *M. sativa* under various growth systems (2): (**A**) Monocropping of *M. sativa*, (**B**) Monocropping of *H. marinum*, (**C**) Parallel intercropping, and (**D**) Mixed system, along with their respective harvest times (3) based on the plant stage (4): incoming flowering stage for *M. sativa* and beginning of the earing stage for *H. marinum*

Each box contained 15 plants, and three modes of cultivation were employed: (i) monoculture of *M. sativa*, (ii) monoculture of *H. marinum*, and (iii) parallel intercropping, which involves one row of *H. marinum* (5 plants) intercropped with two rows of *M. sativa* (10 plants), maintaining a distance of 5 cm between *M. sativa* and *H. marinum* plants within the rows. Additionally, there was mixed cropping, comprising eight plants of *M. sativa* and seven plants of *H. marinum* (Fig. 1). Plants were cultivated under both control treatment and 150 mM NaCl treatment. Three replicates per cultivation mode and treatment were employed, resulting in 24 boxes. Until the harvesting time, which coincided with the initial flowering of alfalfa, corresponding with the kernel extension of *Hordeum marinum*.

After the first harvest, the salt treatment was initiated gradually until the final concentration of 150 mM NaCl was reached, while the control plants were irrigated only with tap water. Plants were harvested three times, the first one before salt treatment and the remaining two after the application of salt.

### Morphological characters measurement

Three forage harvest timings were evaluated, with each harvest, both in sole crops and intercrops, conducted at the start of the earing stage for *H. marinum*. Concurrently, the harvest for *M. sativa* was performed at the initiation of the earing stage, as indicated by Bacchi et al. [30]. Biomass yield was assessed post-harvest for each plot by cutting approximately 5 cm above the ground level. After segregating the plants for analyzing morphological traits across different treatments, they were uprooted, and the soil surrounding the roots was washed away. The plants were then divided into root, stem, and leaf samples. Measurements for each species included the number of axes (NA), length of stems (LS, cm), and root length (LR, cm). Additionally, counts were made for the number of healthy leaves (NHL) and yellow leaves (NYL). The fresh weight of aerial parts and roots was recorded, followed by drying at 60 °C for 48 h in a Memmert UN55 oven, Germany. Subsequently, the dry matter and its constituents (leaves and roots) were determined.

### Plant nutrient analysis

Following the measurement of morphological characters, dried leaves from *M. sativa* and *H. marinum* were utilized for nutrient uptake analysis under various planting systems, both in control conditions and under salt treatment, after each harvest. Dried leaves were ground and passed through a 0.5-mm sieve. A 30 mg portion of each sample was digested with 30 ml of nitric acid (HNO_3_, 0.5%) [31]. After filtration, the digestions were made up to 100 mL and stored at 4 °C. These solutions were then utilized to measure the concentrations of various nutrients, including Na+, K^+^, Ca^2+^, and Mg^2+^. Na^+^ and K^+^ were determined by flame emission spectrophotometry (Flame Photometer 410, Corning), while Ca^2+^ and Mg^2+^ were analyzed using a Varian 220 FS atomic absorption spectrophotometer.

### Soil physico-chemical analyses

All soil samples were collected from each experimental unit to assess residual soil properties, utilizing a soil auger with a 5 cm diameter. Sampling was conducted at a 10 cm interval from 0 to 40 cm soils, and samples from one intercropping plot were obtained by compositing three samples. These soil samples were air-dried and subsequently passed through a 2 mm sieve. Prior to analysis, the samples underwent drying and another round of sieving at 2 mm. Electrical conductivity (CEC) was determined in aqueous extracts at a 1: 1 (w/v) ratio, and soil pH was measured in soil water suspensions using a 1: 2.5 (w/v) ratio. The CEC and pH values were measured using a Crison Conductivimeter and a Crison model 2001 pH-meter, respectively. The percentage of organic matter was determined through direct estimation ignition, while carbon percentage was measured via wet digestion following Kalra and Maynard’s method [31]. Total nitrogen content was analyzed using the Kjeldahl method [32]. For soil phosphorus, the molybdenum blue colorimetric method was applied after persulfate digestion [33]. The saturation percentage was calculated by dividing the volume of water filled in the soil voids by the total volume of the sample, expressed as a percentage. Total calcium carbonate was determined after digestion with acetic acid [34], and potassium (K) was measured after digestion with a 1 M NH4 AcO solution, analyzed using flame emission spectrophotometry (Flame Photometer 410, Corning).

### Statistical analyses

The data from this study were then examined by a two-way analysis of variance (ANOVA). Only variables showing a significant interaction between cropping mode and either crop performance or soil properties were considered in further statistical analysis. The Duncan test at the 5% level was used to compare means. All analyses were performed by using SPSS software (version 20.0 SPSS).

## Results

### Effect of cultivation mode on agronomic traits of *M. sativa* and *H. marinum* under salt-stress

Results from ANOVA showed that the variation of measured traits for both species was explained by the effect of cultivation mode, treatment (control or salinity), harvest period, and their interactions (Table 1).

**Table 1:** Effects of cultivation mode, treatment, number of harvests, and their interactions on measured traits for *M. sativa* and *H. marinum*

	M. sativa															
Trait	Mean	Cultivation mode (CM)		Treatment (T)		Harvest period (H)		CM x T		CM x H		H x T		CM x H x T		
		F	P	F	P	F	P	F	P	F	P	F	P	F	P	
NA	3.98	14.03	0.000	0.718	0.4	5.35	0.007	1.43	0.246	0.77	0.531	0.15	0.701	0.27	0.775	
LS	32.4	60.54	0.000	0.17	0.677	0.89	0.415	5.47	0.006	4.41	0.003	7.04	0.01	6.25	0.003	
LR	9.12	69.6	0.000	5.88	0.018	267.56	0.000	9.25	0.000	17.09	0.000	12.13	0.001	4.69	0.012	
AFW	2.85	70.87	0.000	14.65	0.000	22.71	0.000	2.92	0.061	6.77	0.000	3.15	0.081	8.48	0.001	
ADW	1.95	48.16	0.000	5.05	0.028	20.89	0.000	7.8	0.001	8.09	0.000	1.43	0.237	6.26	0.003	
RFW	1.06	29.21	0.000	2.66	0.108	59.1	0.000	3.65	0.031	2.55	0.047	0.22	0.637	2.7	0.074	
RDW	0.37	27.49	0.000	5.49	0.023	12.44	0.000	2.49	0.09	3.832	0.007	0.7	0.404	4.45	0.015	
NHL	73.73	52.15	0.000	3.56	0.064	19.21	0.000	2.27	0.044	5.53	0.001	0.00	0.995	2.14	0.126	
NYL	17.6	14.02	0.000	12.36	0.001	6.66	0.002	1.86	0.163	1.17	0.332	0.982	0.325	1.98	0.147	
	*H. marinum*															
	Mean	Cultivation mode (CM)		Treatment (T)		Harvest period (H)		CM x T		CM x H		H x T		CM x H x T		
		F	P	F	P	F	P	F	P	F	P	F	P	F	P	
NA	32.28	13.68	0.000	1.02	0.317	7.26	0.001	0.68	0.512	3.98	0.006	4.85	0.031	0.83	0.439	
LS	19.31	4.6	0.014	1.7	0.196	1.19	0.31	3.8	0.053	2.78	0.034	0.88	0.351	0.14	0.872	
LR	8.16	20.64	0.000	27	0.000	378.15	0.000	0.97	0.384	4.81	0.002	2.1	0.152	0.3	0.741	
AFW	4.62	12.57	0.000	2.34	0.131	7.62	0.001	0.56	0.574	1.64	0.176	0.03	0.868	1.93	0.153	
ADW	2.41	13.47	0.000	0.01	0.94	3.75	0.029	0.05	0.95	1.27	0.291	0.52	0.472	1.8	0.174	
RFW	1.07	6.61	0.002	4.13	0.03	23.19	0.000	0.88	0.417	5.72	0.001	1.5	0.226	1.48	0.234	
RDW	0.46	11.67	0.000	15.22	0.000	40.99	0.000	2.99	0.057	7.79	0.000	0.07	0.794	1.18	0.315	
NHL	103.58	25.33	0.000	14.9	0.000	13.67	0.000	3.08	0.053	2.85	0.031	0.04	0.84	7.39	0.001	
NYL	25.38	39.39	0.000	7.82	0.007	82.27	0.000	17.42	0.000	20.36	0.000	0.68	0.412	18.88	0.000	

Number of axes (NA), length of stems (LS. cm), length of roots (LR. cm), aerial fresh weight (AFW. g), aerial dry weight (ADW, g), root fresh weight (RFW, g), root dry weight (RDW. g), number of healthy leaves (NHL) and number of yellow leaves (NYL)

The maximum effect was observed for the cultivation mode. Clear treatment effects were observed on the majority of traits, with significance observed in 5 out of 9 measured for *M. sativa* (root length, aerial fresh weight, aerial dry weight, root dry weight, and number of yellow leaves) and for *H. marinum* (root length, root fresh weight, root dry weight, number of healthy leaves, and number of yellow leaves). Meanwhile, the harvest period had a significant effect for 8 out of 9 traits (not only on LS variation). However, the variations in five out of nine traits, in two out of nine traits, and in five out of nine traits were attributed to the effects of the following interactions: cultivation mode × treatment, cultivation mode × harvest period, and cultivation mode × harvest period × treatment. Overall, under salinity conditions, the variation in agronomic traits was more significantly influenced by the cultivation mode than other factors.

For the first harvest, the highest AFW (3.39 g) and ADW (1.23 g) were obtained for *M. sativa* under the monoculture system while it occurred under the mixed culture mode for *H. marinum* (4.83 g, 2.67 g; 77.85%, 81.65% respectively) (Table S1). In addition, there was an increase of the agronomic traits for *H. marinum* under mixed cropping (MCH) such as LS compared to monoculture of *H. marinum* (MHm); it was by 4.03% meanwhile for parallel intercropping (PIH) it was by 12.57% (Table S1).

For the second harvest, there was an increase in the number of axis (NA), stem length (LS), root length (LR), and aerial dry weight (ADW) under salinity while a decrease was noted for root fresh weight (RFW), root dry weight (RDW), number of healthy leaves (NHL) and number of yellow leaves (NYL) in *M. sativa* under the monoculture system. Furthermore, both parallel intercropping (PI) and mixed cropping (MC) enhanced most growth parameters of *M. sativa* under salinity (Table 2). In addition, the mixed cropping (MC) significantly alleviated the salt effect on *M. sativa*, and resulted in high rise of AFW (11.9%) and ADW (19.01%) compared to monocropping. On the other hand, all agronomical traits were reduced in *H. marinum* under salt stress and this was maintained for the different culture modes. However, under salinity, both parallel intercropping (PI) and mixed cropping (MC) enhanced *H. marinum* biomass parameters, with the highest values observed in the mixed culture mode. For mixed cropping, the increase was 24.06% and 20.45% respectively for AFW and ADW, while for parallel intercropping, it was 17.02% and 10.83% compared to monocropping.

**Table 2:** Comparison of means for measured traits in different cropping systems, under both control and 150 mM NaCl conditions, for two harvests of *M. sativa* and *H. marinum*

	M. sativa							H. marinum					
Trait	Harvest 2			Harvest 3				Harvest 2			Harvest 3		
	MMs	PIS	MCS	MMs	PIS	MCS		MHm	PIH	MCH	MHM	PIH	MCH
		**Control**											
NA	4.33 ± 1.5ab	2.66 ± 0.6b	5.00 ± 1a	5.66 ± 0.6a	3.33 ± 0.6b	5.00 ± 1a		48.33 ± 4.5a	33 ± 4.10a	43.33 ± 2.1a	22.33 ± 2.1a	23.00 ± 6.9a	49.33 ± 1.1 a
LS	44.33 ± 7.6a	29.33 ± 0.6b	27.66 ± 4.6b	48.33 ± 3.2a	23.33 ± 3.5b	21.33 ± 1.5b		23.33 ± 2.9a	18.66 ± 2.9a	20.00 ± 3.5a	24.33 ± 0.6a	16.33 ± 2.1 b	15 ± 1b
LR	14 ± 1.7a	6.33 ± 1.5b	10.33 ± 2.5a	20.33 ± 1.2a	8 ± 1.7c	13.00 ± 2.6b		8.67 ± 2.3a	9 ± 1.7a	12.33 ± 3.1 a	13.33 ± 1.2a	11.00 ± 1ab	13 ± 1b
AFW	4.81 ± 0.5a	0.94 ± 0.1c	2.1 ± 0.7b	2.2 ± 0.3a	0.94 ± 0b	1.12 ± 0.2b		4.53 ± 0.5b	4.03 ± 1.1b	7.96 ± 0.9a	2.77 ± 0.6c	7.52 ± 0.6a	5.88 ± 0.5b
ADW	2.91 ± 0.1a	0.44 ± 0.1c	1.04 ± 0.4b	1.31 ± 0.4a	0.51 ± 0b	0.58 ± 0.2b		2.2 ± 0.3b	1.9 ± 0.5b	3.87 ± 0.2a	1.51 ± 0.5b	3.28 ± 0.6a	2.8 ± 0.3a
RFW	0.99 ± 0.1a	0.64 ± 0.2b	0.3 ± 0.1c	2.23 ± 0.6a	1 ± 0b	2 ± 0a		1.24 ± 0.3a	1.56 ± 0.7a	1.03 ± 0.2a	0.33 ± 0.6b	2 ± 1ab	3.33 ± 1.2a
RDW	0.37 ± 0.1ab	0.43 ± 0.1a	0.19 ± 0.1b	1 ± 0a	0 ± 0b	0.33 ± 0.6b		0.7 ± 0.2a	0.98 ± 0.4a	0.65 ± 0.1a	0.24 ± 0b	0.84 ± 0a	1.15 ± 0a
NHL	133 ± 2.3a	47.33 ± 5.5c	99 ± 5b	62 ± 7a	63.33 ± 7.5a	0 ± 0b		157.33 ± 5.14a	82.66 ± 5.19b	149 ± 4.2a	76.33 ± 2.14b	163.33 ± 2.37a	149.33 ± 4.3a
NYL	1.67 ± 2.9b	10 ± 3a	5.66 ± 1.5ab	10.33 ± 2.1b	12.33 ± 3.5b	42.33 ± 3.5a		11.33 ± 1.5b	19.66 ± 6.1b	31.66 ± 5.5a	19.33 ± 3.2b	6.33 ± 0.6b	81.33 ± 2.5a
		**NaCl**											
NA	3.66 ± 1b	2.66 ± 0.5b	5.17 ± 1a	4 ± 1.2b	3.5 ± 1.9b	5.16 ± 1.5a		24.5 ± 5.1 a	26.5 ± 5.2a	39.16 ± 8.9a	27.16 ± 2.1b	27.83 ± 4.9b	52.5 ± 2.1a
LS	39.17 ± 4.7a	27.5 ± 5.8b	22.66 ± 4.9b	42.33 ± 6.2a	23.5 ± 5.4b	43.66 ± 7.1a		17.5 ± 2.4a	17.83 ± 4.3a	19.5 ± 5.7a	20.17 ± 3.6a	16.33 ± 2.6a	18 ± 3a
LR	12.75 ± 2.5a	5.83 ± 1.1 b	13.66 ± 3.7a	13.17 ± 1.5a	11 ± 1.8b	8.33 ± 1.5c		8.16 ± 2.4b	6.58 ± 1.3b	10.83 ± 2.1a	10.83 ± 1.2a	7.83 ± 0.8b	10.83 ± 1.5a
AFW	4.96 ± 2a	1.92 ± 0.4b	5.63 ± 1.6a	3.32 ± 0.3a	1.39 ± 0.4b	1.04 ± 0.4b		3.85 ± 2a	4.65 ± 3.1a	5.07 ± 2.6a	3.12 ± 0.9a	5.24 ± 1.2a	5.46 ± 3.2a
ADW	2.3 ± 1.2a	0.64 ± 0.2b	2.84 ± 0.7a	1.35 ± 0.1a	0.7 ± 0.2a	0.77 ± 0.3b		2.14 ± 1.1 a	2.4 ± 1.7a	2.69 ± 1.3 a	1.63 ± 0.9b	2.9 ± 1.1a	3.66 ± 0.9a
RFW	1.71 ± 0.9a	0.27 ± 0.1b	0.85 ± 0.4b	2.29 ± 1.3a	1.07 ± 0.4b	1.63 ± 0.5b		0.84 ± 0.3a	1.43 ± 1.9a	0.88 ± 0.7a	0.43 ± 0.5a	1.34 ± 1.2ab	1.71 ± 1a
RDW	1 ± 0.4a	0.14 ± 0.1c	0.56 ± 0.3b	1.07 ± 0a	0.29 ± 0.4c	0.58 ± 0.5b		0.42 ± 0.2a	0.36 ± 0.1a	0.59 ± 0.5a	0.18 ± 0a	0.57 ± 0.5a	0.62 ± 0.5a
NHL	166.33 ± 6.6a	56.33 ± 3.3b	103.33 ± 4.9b	102 ± 2.3a	27.66 ± 6.5b	42.66 ± 6b		50.33 ± 8b	94.66 ± 2.4a	123.83 ± 7.7a	47.33 ± 3.1a	75.16 ± 5.5a	158.33 ± 7.1a
NYL	15 ± 5.10b	15.33 ± 4.1b	50.66 ± 6.1a	13.16 ± 5.8b	31.83 ± 8ab	55.66 ± 2.1b		20.33 ± 5.8a	21.16 ± 6.1a	35.66 ± 3a	34 ± 3.1a	48 ± 1.1b	51.66 ± 2.1b

Mean ± Standard Deviation (SD). Number of axes (NA), length of stems (LS, cm), length of roots (LR, cm), aerial fresh weight (AFW, g), aerial dry weight (ADW, g), root fresh weight (RFW, g), root dry weight (RDW, g), number of healthy leaves (NHL) and number of yellow leaves (NYL). Cropping mode investigated: mono-culture of *M. sativa* (Mms), *M. sativa* in parallel intercropping (PIS), *M. sativa* in mixed culture (MCS). Means followed by the same letter(s) or common letters are not significantly different among the cultivation modes for each trait according to Duncan test at 5%

For the third harvest, there was a reduction for most growth parameters, except NHL and NYL in *M. sativa* under the monoculture system, whereas the highest levels of the traits were registered under monoculture of *M. sativa (*MMS) (Table 2). The mixed culture mode improved the growth and biomass parameters in *H. marinum* and mitigated the salt stress effects, resulting in the highest rise of NHL (70.10%), AFW (42.86%), ADW (55.46%) compared to monocropping (Table 2).

### Effect of cultivation mode on nutrient acquisition of *M. sativa* and *H. marinum* under salt stress

Results from ANOVA showed that the variation in nutrient acquisition was explained by the effects of cultivation mode, treatment, harvest period, and their interactions (cultivation mode x treatment; cultivation mode x harvest period; treatment x harvest; and cultivation mode x treatment x harvest period) (Table 3). A significant difference was noted for all analyzed minerals (except for the potassium) measured under different cultivation modes. Furthermore, the treatment had a significant effect only on the variation in the ratio Na^+^/K^+^ (*P* ≤ 0.05). The harvest period had a significant effect on the variation in the nutrient acquisition except for potassium. Additionally, the interaction cultivation mode x harvest period had a high significant effect on all nutrient trait’s variation.

**Table 3:** ANOVA analysis of the variation in cultivation mode, treatment, number of harvests, and their interactions on plant nutrient acquisition

Trait	Mean	Cultivation mode (CM)		Treatment (T)		Harvest period (H)		CM x T		CM x H		H x T		CM x H x T	
		F	P	F	P	F	P	F	P	F	P	F	P	F	P
Na^+^	2.42	15.21	0.000	132.39	0.000	96.75	0.000	11.16	0.000	10.34	0.000	1.52	0.225	4.54	0.008
K^+^	2.26	1.77	0.169	4.99	0.031	0.275	0.761	3.59	0.022	3.4	0.009	4.24	0.046	3.44	0.026
K^+^/ Na^+^	1.05	8.80	0.000	12.11	0.001	142.18	0.000	0.85	0.472	6.33	0.000	0.76	0.389	0.60	0.616
Ca^2+^	22.42	37.18	0.000	1.87	0.179	71.78	0.000	6.19	0.001	45.10	0.000	22.36	0.001	18.38	0.000
Mg^2+^	1.46	32.33	0.000	0.32	0.576	9.6	0.000	10.1	0.000	22.99	0.000	12.97	0.001	9.01	0.000
Ca^2+^/Mg^2+^	15.56	3.85	0.016	0.31	0.579	30.83	0.000	2.57	0.067	20.3	0.000	0.95	0.337	3.67	0.02

F: Snedecor-Fisher coefficient. Significant (*P* ≤ 0.05); not significant (*P* > 0.05)

The cultivation mode significantly affected the nutrient acquisition in the shoots of both species grown under the monoculture mode and the total nutrient acquisition resulting from the intercropping of the two species in the two distinct modes (Table S2). For the first harvest, the cultivation system has no significant impact on sodium and potassium contents. However, it had an effect on magnesium and calcium contents, where their highest values, namely 17.8 mg/kg MS and 2.14 mg/kg MS, respectively, were noted in *M. sativa* under the monoculture mode.

**Fig. 2:**
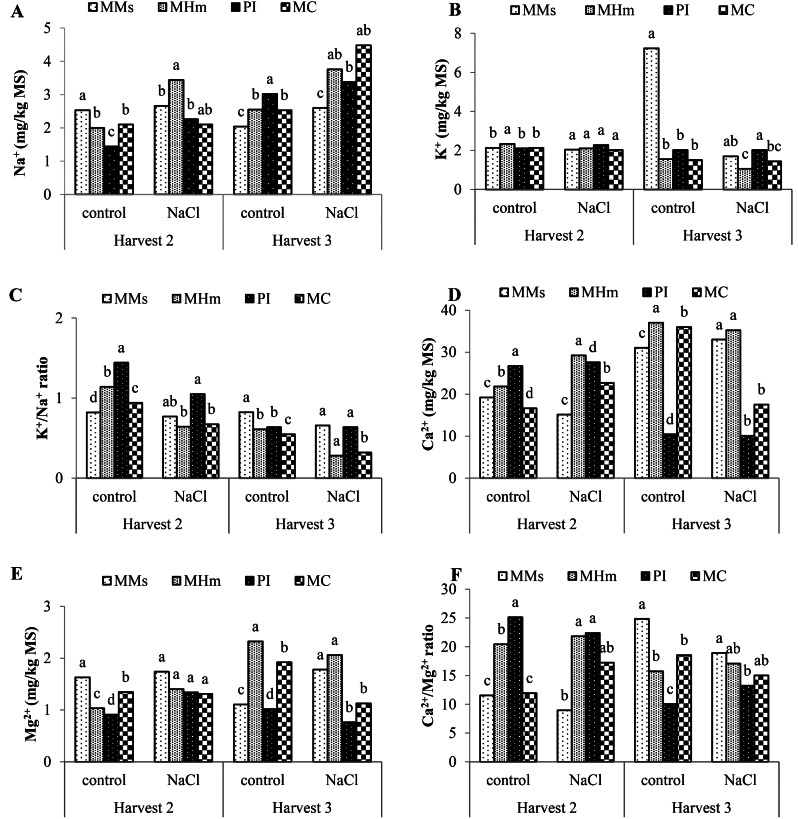
Mean comparison of measured nutrient acquisition (**A**: sodium (Na^+^); **B**: potassium (K^+^); **C**: potassium / sodium ratio (K^+^/Na^+^); **D**: calcium (Ca^2+^); **E**: magnesium (Mg^2+^); **F**: calcium/magnesium ratio (Ca^2+^/Mg^2+^)) during two harvests under control treatment (non-saline) and salinity treatment within different growing systems. The cropping modes investigated were monoculture of M. sativa (MMs), monoculture of H. marinum (MHm), and parallel intercropping (PI), and mixed cropping (MC). Means followed by the same letter(s) or common letters are not significantly different among the cultivation modes for each parameter according to Duncan test at 5%

For the second harvest, under the control treatment, an increase in K^+^/Na^+^ ratio and a decrease in Ca^2+^/Mg^2+^, were noted for both species under the monoculture mode compared to the first harvest (Table 4; Fig. 2). The highest effect of salinity on Na^+^ and Mg^2+^contents was observed under monoculture of *M. sativa* (MMs), for Ca^2+^ in the parallel intercropping (PI) and for K^+^ in the monoculture of *H. marinum* (MHm) (Fig. 2). Thus, the salt stress reduced these two ratios in monoculture of *M. sativa* (MMs) while it increased them in monoculture of *H. marinum* (MHm). However, the parallel intercropping (PI) alleviated the nutrient uptake impairment under salt-stress, as noted by an increase of K^+^/Na^+^ by 26.67% and 36.19% compared to monoculture of *M. sativa* and *H. marinum*, respectively. In addition, Ca^2+^/Mg^2+^ ratios rise by 60% and 2.41% compared to monoculture of *M. sativa* and *H. marinum*.

**Table 4:** Means comparison of measured nutrient acquisition during 2 harvests under control treatment (non-saline) and salinity treatment within different growing systems

	Harvest 2					Harvest 3							
	MMs	MHm	PI	MC		MMs		MHm		PI		MC	
	Control												
Na^+^	2.53 ± 0.4a	2.00 ± 0.01b	1.44 ± 0.01c	2.10 ± 0.1b			2.04 ± 0.04c		2.55 ± 0.05b		3.02 ± 0.02a		2.53 ± 0.03b
K^+^	2.13 ± 0.02b	2.33 ± 0.1a	2.10 ± 0.1b	2.13 ± 0.01b			7.23 ± 3.4a		1.55 ± 0.05b		2.02 ± 0.02b		1.51 ± 0.01b
K^+^/ Na^+^	0.82 ± 0.01d	1.14 ± 0.02b	1.44 ± 0.01a	0.94 ± 0.01c			0.82 ± 0.03a		0.61 ± 0.01b		0.64 ± 0.04b		0.55 ± 0.05c
Ca^2+^	19.22 ± 0c	21.82 ± 0b	26.71 ± 0.01a	16.64 ± 0.01d			31.04 ± 0.04c		37.04 ± 0.04a		10.42 ± 0.02d		36 ± 0.05b
Mg^2+^	1.63 ± 0a	1.03 ± 0c	0.91 ± 0.02d	1.35 ± 0.01b			1.107 ± 0.005c		2.32 ± 0.03a		1.017 ± 0.02d		1.92 ± 0.02b
Ca^2+^/Mg^2+^	11.54 ± 0c	20.43 ± 0b	25.14 ± 4a	11.93 ± 0c			24.84 ± 0.03a		15.75 ± 0.05b		10.05 ± 0.05c		18.54 ± 0.04b
	**NaCl**												
Na^+^	2.66 ± 0.1b	3.44 ± 0.7a	2.26 ± 0.3b	2.1 ± 0ab			2.6 ± 0.12c		3.76 ± 0.28ab		3.38 ± 0.7b		4.48 ± 0.2a
K^+^	2.04 ± 0.1a	2.1 ± 0.1a	2.28 ± 0.4a	2.02 ± 0a			1.7 ± 0.02ab		1.06 ± 0.3c		2.02 ± 0.3a		1.44 ± 0.2bc
K^+^/ Na^+^	0.77 ± 0.1ab	0.64 ± 0.2b	1.05 ± 0.3a	0.673 ± 0b			0.66 ± 0.04a		0.28 ± 0.06b		0.64 ± 0.25a		0.32 ± 0.04b
Ca^2+^	15.14 ± 3.8c	29.24 ± 2a	27.6 ± 1.6ab	22.68 ± 2.7b			33 ± 4.8a		35.24 ± 4.44a		10.1 ± 0.94b		17.52 ± 3.8b
Mg^2+^	1.74 ± 0.1a	1.40 ± 0.3a	1.34 ± 0.4a	1.31 ± 0a			1.78 ± 0.14a		2.06 ± 0.06a		0.76 ± 0.01b		1.13 ± 0.42b
Ca^2+^/Mg^2+^	8.94 ± 2.9b	21.82 ± 4.5a	22.38 ± 3.5a	17.24 ± 1.5ab			18.91 ± 4.2a		17.08 ± 1.65ab		13.24 ± 1.14b		15.01 ± 1.49ab

Mean ± Standard Deviation (SD). Cropping mode investigated: monoculture of *M. sativa* (MMs), monoculture of *H. marinum* (MHm), parallel intercropping (PI), mixed cropping (MC). Means followed by the same letter(s) or common letters are not significantly different among the cultivation modes for each parameter according to Duncan test at 5%

For the third harvest, under the control treatment, the highest content of Na^+^ (3.02 mg/kg MS) and K^+^ (2.02 mg/kg MS) were found in the parallel intercropping (PI) and monoculture of *M. sativa* (MMs), respectively, while monoculture of *H. marinum* (MHm) exhibited the highest levels of Mg^2+^ (37.04 mg/kg MS) and Ca^2+^ (2.32 mg/kg MS). Under salt stress, the mixed cropping (MC) improved the uptake of Na^+^ by 37.80% and 10.05% compared to MMs and MHm, respectively. While the parallel intercropping (PI) improved the uptake of K^+^ by 15.84% and 47.52% compared to MMs and MHm, respectively, whereas the highest contents of Ca^2+^ and Mg^2+^ were found in the monoculture of *M. sativa* (MMs) and monoculture of *H. marinum* (MHm), respectively.

### Effect of cultivation mode on soil chemical properties in *M. sativa* and *H. marinum* under salt stress

The variation in soil chemical properties was explained by the effects of cultivation mode, treatment, harvest period and their interactions: (cultivation mode x treatment), (cultivation mode x harvest period) and (treatment x harvest period) (Table 5). A significant variation was observed for most parameters among cultivation modes and treatments. Furthermore, the variation in soil fertility was explained by the interaction cultivation mode x treatment.

**Table 5:** Significance levels of cultivation mode, treatment, and the interaction between cultivation mode and treatment for different physicochemical properties of the soil

Trait	Mean	Cultivation mode (CM)		Treatment (T)		Interaction (CM x T)	
		F	P	F	P	F	P
pH	8.25	3.413	0.043	234.189	0.000	9.523	0.001
CEC	11.92	7.622	0.002	49.638	0.000	3.859	0.030
S	35.75	4.000	0.027	0.000	1.000	12.000	0.000
OM	3.24	18.509	0.000	8.265	0.011	2.868	0.069
C	1.88	18.469	0.000	9.114	0.008	2.947	0.064
N	11.41	26.211	0.000	4.636	0.047	5.455	0.009
P_2_O_5_	14.91	2.446	0.101	2.253	0.153	3.310	0.047
K_2_O	305.57	119.625	0.000	17.924	0.001	40.178	0.000
CaCo_3_	2.69	6.333	0.005	9.000	0.008	33.000	0.000

F: coefficient of Snedecor-Fisher significant (*P* ≤ 0.05). Potential of hydrogen (pH), cation exchange capacity (CEC. mS/cm), percentage of soil saturation (S), percentage of organic matter (OM. %), percentage of total carbon (C. %), total nitrogen (N. mg, g Ms), total phosphorus (P_2_O_5_. mg.g Ms), total potassium (K_2_O. ppm), percentage of total Calcium Carbonate (CaCO_3_. %)

Under the control treatment, there was a reduction of the percentage of total CaCO_3_ (1%) under mixed cropping compared to other modes (3%). However, an increase of the percentage of organic matter (OM) was found under the intercropping system with highest increment (41.58%) was observed with MCH compared to its monoculture. An increase in pH and total phosphorus were noted under the parallel intercropping. The highest electrical conductivity (CEC) (13.6 mS/cm) and soil saturation (38%) were noted in the monoculture of *H. marinum* (MHm) while the highest total nitrogen value was registered for monoculture of *M. sativa* (MMs). Under salt stress, there was an increase of the pH, CEC, OM and C, while a decrease of the total nitrogen (N), P_2_O_5_ and K_2_O occurred in both the monoculture of *M. sativa* (MMs) and the monoculture of *H. marinum* (MHm). However, under the intercropping mode, there was a reduction in the pH and the CEC of the soil, with the lowest values of 8.37and 13.55 mS/cm, respectively, being noted in parallel intercropping (PI) and mixed cropping (MC). In addition, the mixed cropping (MC) alleviated salt-stress resulted in the highest percentage of OM (4.12%); C (2.39%) and P_2_O_5_ (50.59 ppm) (Table 6).

**Table 6:** Comparison of mean nutrient acquisition in different cropping systems under both control and 150 mM NaCl conditions. The values represent the means of three harvests

	MMs	MHm	PI	MC
	Control			
pH	8.11 ± 0.02b	8.01 ± 0.01c	8.21 ± 0.01a	8.01 ± 0.01c
CEC	6.72 ± 0.01d	13.60 ± 0.03a	8.16 ± 0.01c	10.11 ± 0.01b
S	35 ± 0b	38 ± 0a	35 ± 0b	35 ± 0b
OM	2.36 ± 0.01d	3.21 ± 0.02b	2.61 ± 0.01c	4.04 ± 0.03a
C	1.35 ± 0.01d	1.85 ± 0.03b	1.51 ± 0.02c	2.336 ± 0.025a
N	16.22 ± 0.03a	8.93 ± 0.02d	11.73 ± 0.03b	10.62 ± 0.02c
P_2_O_5_	9.61 ± 0.015c	11.02 ± 0.01b	12.81 ± 0.02a	7.44 ± 0.04d
K_2_O	130 ± 0c	112.25 ± 0.06d	357 ± 0.25b	780.08 ± 0.01a
CaCO_3_	3 ± 0a	3.01 ± 0a	3 ± 0a	1 ± 0b
	**NaCl**			
pH	8.43 ± 0.09a	8.48 ± 0.01a	8.37 ± 0.04a	8.39 ± 0.11a
CEC	13.43 ± 0.7a	15.08 ± 2.22a	14.72 ± 2.12 a	13.55 ± 3.22a
S	35 ± 1.5a	35 ± 0a	36.5 ± 0a	36.5 ± 1.5a
OM	3.20 ± 0.27b	3.12 ± 0.33b	3.3 ± 0.79ab	4.12 ± 0.24a
C	1.86 ± 0.19b	1.80 ± 0.16b	1.92 ± 0.46ab	2.39 ± 0.14a
N	12.6 ± 1.82a	9.38 ± 1.96b	10.5 ± 0.14ab	11.34 ± 1.26ab
P_2_O_5_	6.58 ± 3.35b	6.24 ± 0.18b	14.95 ± 0.18ab	50.59 ± 3.11a
K_2_O	116 ± 5.27c	235.25 ± 4bc	352.5 ± 5.9ab	361.5 ± 5.12a
CaCO_3_	2.5 ± 0.57b	2.5 ± 0b	3 ± 0.5ab	3.5 ± 0.5a

Mean ± Standard Deviation (SD). Parameters measured were: potential of hydrogen (pH), cation exchange capacity (CEC, mS/cm), percentage of soil saturation (S), percentage of organic matter (OM, %), percentage of total carbon (C, %), total nitrogen (N, mg, g Ms), total phosphorus (P_2_O_5_, mg.g Ms), total potassium (K_2_O, ppm), percentage of total Calcium Carbonate (CaCO_3_, %). Cropping mode investigated were monoculture of *M. sativa* (MMs), monoculture of *H. marinum* (MHm), parallel intercropping (PI), mixed cropping (MC). Means followed by the same letter(s) or common letters are not significantly different among the cultivation modes for each trait according to Duncan test at 5%

## Discussion

Intercropping contributes significantly to mitigating salinity conflicts in arid areas involving legume and grass crops. Legumes, known for their exceptional biological nitrogen fixation capabilities, traditionally serve as valuable green manure resources that enhance the growth of companion crops, as highlighted by Enrico et al. [35]. In this study, *M. sativa* demonstrated the highest biomass development under monoculture, while the mixed intercropping mode with *M. sativa* improved *H. marinum* development, resulting in increased AFW, ADW, and LS. In concordance with our findings, intercropping with legumes has been shown to enhance yields and overall output, establishing it as a favored cropping approach to replace traditional monocropping, as indicated by Qing et al. [36]. Additionally, as explained by Hu et al. [15], under mono intercropping (MC) conditions, the limited space for root extension in both species hindered the formation of root nodules in our case, particularly in alfalfa seedlings. This limitation contributed to a reduced capacity for nitrogen fixation. This, in turn, intensifies the competition for nutrients between alfalfa and *H. marinum* in mixed intercropping systems. Notably, cereals, such as *H. marinum*, are recognized for their higher nutrient competitiveness compared to Leguminosae [24]. Consequently, the intercropping of alfalfa with *H. marinum* is seen to enhance the latter’s development and growth. Salt stress significantly impacted forage yield in both species, resulting in a notable decline in fresh and dry plant weights, with *M. sativa* exhibiting lower susceptibility. These results are consistent with observations made by Yu et al. [37], indicating that *M. sativa* demonstrates moderate salinity tolerance compared to other forage crops, and among legumes, it displays a high level of salt tolerance compared to wheat and maize [22, 27, 38]. In addition, Isayenkov et al. [39] described *H. marinum* as a halophyte, which is considered one of the main genetic sources for salinity tolerance. Our results demonstrated a significant positive effect of *M. sativa* intercropping on crop biomass. Furthermore, it was observed that the mixed cropping system had the most substantial impact on the total yield of crop plants grown under salinity. Indeed, in intercropping systems, plants play a direct or indirect role in regulating and modifying soil properties during salt stress, as highlighted by Mishra and Singh [40]. Particularly, *M. sativa* can mitigate salt accumulation by limiting heat, air, and water vapor exchange between the soil and the atmosphere [17]. The root interaction between the two species in intercropping systems may have restricted each other’s growth. Halophyte roots have their adaptive strategy-related responses to saline soil, and physiological responses to sea barley may also exist. A higher salt content in *M. sativa* roots influences sea barley’s belowground growth and suppresses shoot biomass [41]. Several studies have shown that yield per unit of land is increased in a mixed system rather than a monoculture [42, 43]. Therefore, the application of salt treatment had a noticeable impact in the combined cropping system for *H. marinum* and *M. sativa*, particularly during the third harvest. This impact may be due to leaf damage caused by salt accumulation over time, particularly in older leaves, leading to early leaf senescence [44]. Similar results were observed for *M. sativa* intercropped with maize [24].

Nutrient uptake stands out as one of the most limiting factors for crop production altered by salt stress. In this study, salt stress affects nutrient uptake in both species. The cultivation systems also significantly influenced nutrient uptake in the shoots of each species when grown individually. Furthermore, the combined nutrient uptake is influenced by the intercropping methods used. Horchani et al. [45] showed that salt stress affects nutrient uptake by limiting water absorption and disrupting the plant’s ability to absorb nutrients. In the current study, salt stress reduced these in K^+^/Na^+^ and Ca^2+^/Mg^2+^ in *M. sativa* monoculture (MMs) while it increased them in *H. marinum* monoculture (MHm). These results suggest that intercropping, especially with *M. sativa*, had a stronger ability to remove Na^+^ compared to *H. marinum* from the soil. However, parallel intercropping (PI) and mixed intercropping (MC) alleviated the impairment of nutrient uptake under salt stress, particularly during the third harvest. This effect could be attributed to the accumulation of sodium, caused by prolonged exposure to salt stress, and Ca^2+^ release in the soil. Studies have shown that Ca^2+^ released from the soil promotes the removal of Na^+^ from cation exchange sites. Therefore, Na^+^ content is often negatively correlated with Ca^2+^ content in soils [46]. Similarly, Ghaffarian et al. [47] showed that the intercropping of different species in the presence of halophyte plants in saline conditions leads to a significant uptake of sodium from the soil. The effect of intercropping on potassium uptake was not evident, except during the third harvest, where higher values were recorded in the parallel intercropping method. This result can be explained by the fact that salinity inhibits the absorption of potassium due to the increased concentration of sodium and its competitive effect on potassium uptake [48].

Furthermore, our study revealed that the cultivation system significantly affects soil properties, resulting in a reduction in pH and electrical conductivity (CEC), while increasing organic matter and carbon content, especially in mixed culture. Soil salt accumulation due to evapotranspiration surpasses the removal of salt through leaching and absorption during the growth of *H. marinum*, consequently affecting CEC. This phenomenon is attributed to the increased release of H^+^ ions resulting from biological nitrogen fixation, root secretions, and the dephosphorylation of organic phosphorus when intercropped with *M. sativa*. Consequently, there is an enhancement in soil pH conditions. Furthermore, the heightened concentration of H^+^ ions and the increased activity of alkaline phosphatase in the soil contribute to the improvement of carbon content [49]. The current findings indicated a decrease in Na^+^, K^+^ uptake, and pH with mixed cropping of *H. marinum* compared to monoculture system. These changes can be linked to the diverse absorption of ions by crops, interactions among ions, and variations in ion solubility. Intercropping with *M. sativa* improves soil quality by reducing evaporation and salinization, and enhancing soil fertility through the production of organic acids [50]. The presence of leguminous crops in intercropping systems increases nitrogen availability in the soil. *M. sativa*, with its deep and strong root system, plays a significant role in improving soil structure and reducing soil density [50]. Similar observations were revealed in intercropping *M. sativa*/cotton [17] and *M. sativa*/wheat [18]. Also, Liang and Shi’s [17] study on intercropping of *M. sativa* with cotton showed an increase in salt removal and organic carbon content and an improvement in soil porosity. A study by Cong et al. [51] supports our findings and demonstrates that intercropping has the potential to provide numerous benefits to the agro-ecosystem, such as increased yields, improved soil quality, and soil carbon sequestration.

Overall, our results highlight the importance of intercropping as a cultivation method for promoting and maintaining soil health. This finding underscores the significance of adopting intercropping as a sustainable agricultural practice. A noteworthy observation was made regarding total nitrogen in our study, as a significant effect was observed not only in the monoculture of *M. sativa* but also in the other two intercropping systems. This can be attributed to the fact that *M. sativa* is a leguminous crop, which has the ability to fix nitrogen in the soil.

## Conclusion

In this study, we explored the effects of *M. sativa*/*Hordeum marinum* intercropping on soil alkali-salinity in contrast to traditional sole culture. The findings revealed positive outcomes where intercropping exhibited beneficial effects on crop growth and soil quality in saline conditions. Notably, our investigation highlighted the substantial impact of mixed cropping, particularly evident at the second harvest, significantly benefiting *H. marinum*. Intercropping notably reduced soil salinity, pH levels, and the content of major ions such as Ca^2+^ and Mg^2+^, underscoring its effectiveness in mitigating the adverse effects of salt stress on *H. marinum* production and soil fertility.

Additionally, the choice of cultivation system significantly influenced nutrient uptake in both species’ shoots and contributed to improved soil physicochemical properties under salt treatment. In conclusion, advocating for *M. sativa*/*H. marinum* systems as a long-term agronomic amendment to enhance soil salinity appears well-founded. However, further investigations focusing on analyzing the bacterial community structure within diverse intercropping systems under both treatments are warranted to deepen our understanding of these agricultural practices.

### Electronic supplementary material

Below is the link to the electronic supplementary material.

**Supplementary Material 1**: **Table S1**. Mean comparison of measured traits in different cropping systems under control treatment in the first harvest of M. sativa and H. marinum. **Table S2**. Mean comparison of measured nutrient acquisition during the first harvest under control treatment (non-saline) within different growing systems.

## Data Availability

The plant material consists of the variety Gabès 2353 of alfalfa, which is currently marketed in Tunisia, and the Kelbia 4 line of *Hordeum marinum*, which has been developed by Prof. Mounawer Badri’s team. A quantity of seeds from each genotype is conserved by the Forage Species Bank at the Centre of Biotechnology of Borj Cedria (CBBC) (http://www.cbbc.rnrt.tn/) and can be provided upon request. All data generated or analyzed during this study are included in this published article and its supplementary information files.
