# Supplementary material for: Improving productivity and soil fertility in Medicago sativa and Hordeum marinum through intercropping under saline conditions
Source: BMC Plant Biol. 2024 Mar 1;24:158. doi: 10.1186/s12870-024-04820-3 (PMC10905945; doi:10.1186/s12870-024-04820-3)
Supplement: Supplementary file 1 — Supplementary Material 1: Table S1. Mean comparison of measured traits in different cropping systems under control treatment in the first harvest of M. sativa and H. marinum. Table S2. Mean comparison of measured nutrient acquisition during the first harvest under control treatment (non-saline) within different growing systems. [file 12870_2024_4820_MOESM1_ESM.docx]

**Supplementary Table 1.** Means comparison of measured traits in different cropping systems in control treatment in the first harvest of *M. sativa* and *H. marinum*

| Trait | *M. sativa* | | |  | *H. marinum* | | |
| --- | --- | --- | --- | --- | --- | --- | --- |
|  | MMs | PIS | MCS |  | MHm | PIH | MCH |
| AN | 4.22 ± 1.3b | 2.33 ±1.7b | 3.11 ± 1.3ab |  | 9.11 ± 3.1b | 29.89 ± 5.1a | 28 ± 6.5a |
| LS | 42.11 ±5.5a | 19.67 ± 6.6b | 31.11 ±4.2 b |  | 21.22 ± 5.2a | 19.33 ± 4.3a | 22.11 ± 2a |
| AFW | 3.39 ± 0.8a | 0.59 ± 0.3c | 2.02 ± 0.7b |  | 1.07 ± 0.4a | 3.33 ± 1.6a | 4.83 ± 3a |
| ADW | 1.23 ± 0.3a | 0.25 ± 0.2b | 1.04 ± 0.6a |  | 0.49 ± 0.2b | 1.9 ± 1a | 2.67 ± 1.8a |
| NHL | 23.33 ± 6.34a | 61.22 ± 9.45c | 118.44 ± 7.43b |  | 47.78 ± 5.8b | 71.67 ± 9.6b | 106.67 ± 9.7a |

Mean ± Standard Deviation (SD). Number of axes (AN), length of stems (LS, cm), aerial fresh weight (AFW, g), aerial dry weight (ADW, g) and number of healthy leaves (NHL). Cropping mode investigated were mono-culture of *M. sativa* (Mms), *M. sativa* in parallel intercropping (PIS), *M. sativa* in mixed culture (MCS), mono-culture of *H. marinum* (MHm), parallel intercropping of *H. marinum* (PIH) and mixed cropping of *H. marinum* (MCH). Means followed by the same letter(s) or common letters are not significantly different among the lines for each trait according to Duncan test at 5%.

**Supplementary Table 2.** Means comparison of measured nutrient acquisition during the first harvest under control treatment (non-saline) within different growing systems

|  | Harvest 1 | | | |
| --- | --- | --- | --- | --- |
|  | MMs | MHm | PI | MC |
| Na^+^ | 0.83 ±0.1a | 1.2 ±0.5a | 1.33 ±0.1a | 1.2 ±0.1a |
| K^+^ | 2.43 ±0.2a | 2.51 ±0.1a | 2.43 ±0.4a | 2.15 ±0.1a |
| K^+^/ Na^+^ | 2.96 ±0.3a | 2.11 ±0.2ab | 2.027 ±0.9ab | 1.81 ±0.3b |
| Ca^2+^ | 17.8±0.8a | 15.36±0.1b | 11.58±0.3c | 14.15±2.2b |
| Mg^2+^ | 2.14±0.1a | 1.37±0.3b | 1.43±0.3b | 1.45±0.3b |
| Ca^2+^/mg^2+^ | 8.333±0.2a | 11.58±2.5a | 8.31±1.4a | 10.08±2.9a |

Mean ± Standard Deviation (SD). Cropping mode investigated were monoculture of *M. sativa* (MMs), monoculture of *H. marinum* (MHm), parallel intercropping (PI) and mixed cropping (MC). Means followed by the same letter(s) or common letters are not significantly different among the lines for each parameter according to Duncan test at 5%.
